# Promotion of natural tooth repair by small molecule GSK3 antagonists

**DOI:** 10.1038/srep39654

**Published:** 2017-01-09

**Authors:** Vitor C. M. Neves, Rebecca Babb, Dhivya Chandrasekaran, Paul T. Sharpe

**Affiliations:** 1 Department of Craniofacial Development and Stem Cell Biology, Dental Institute, Kings College London, UK

## Abstract

The restoration of dentine lost in deep caries lesions in teeth is a routine and common treatment that involves the use of inorganic cements based on calcium or silicon-based mineral aggregates. Such cements remain in the tooth and fail to degrade and thus normal mineral volume is never completely restored. Here we describe a novel, biological approach to dentine restoration that stimulates the natural formation of reparative dentine via the mobilisation of resident stem cells in the tooth pulp. Biodegradable, clinically-approved collagen sponges are used to deliver low doses of small molecule glycogen synthase kinase (GSK-3) antagonists that promote the natural processes of reparative dentine formation to completely restore dentine. Since the carrier sponge is degraded over time, dentine replaces the degraded sponge leading to a complete, effective natural repair. This simple, rapid natural tooth repair process could thus potentially provide a new approach to clinical tooth restoration.

Dentine is a vital tooth mineral that is produced by highly specialised mesenchymal cells called odontoblasts. When tooth mineral is compromised either following trauma or infection (caries), the inner cellular soft pulp tissue can become exposed to the external environment and become infected. Clinical repair of tooth damage currently involves the use of mineral aggregates that are used to fill the space in dentine created following removal of decay or trauma^1^,^2^,^3^,^4^,^5^. When the soft inner pulp tissue is exposed, a natural repair process is activated that involves the mobilisation of resident mesenchymal stem cells to differentiate into new odontoblast-like cells that secrete a form of tertiary (reparative) dentine^6^,^7^,^8^,^9^. The reparative dentine produced forms a thin band of dentine (dentine bridge) that serves to protect the pulp from infection by sealing the tooth pulp from the external environment. Unfortunately, natural reparative dentine formation is insufficient to effectively repair large lesions, such as those involving the loss of dentine after caries removal and hence artificial mineral aggregates are used to fill the tooth and replace the lost dentine.

The activation of Wnt/β-cat signalling is an immediate early response to tissue damage and appears to be essential for stimulating the cellular-based repair in all tissues^10^,^11^,^12^,^13^. Axin 2 is a negative regulator and also a downstream target of this signaling pathway. A key cytoplasmic component of Wnt/β-cat signal transduction is the enzyme, glycogen synthase kinase 3 (GSK-3) that in the absence of Wnt ligand/receptor binding, phosphorylates β-catenin and Axin leading to ubiquitination and degradation. In the presence of Wnt ligands, GSK-3 activity is inhibited allowing β-catenin to enter the nucleus where it interacts with Lef/Tcf transcription factors to regulate expression of target genes, that include Axin2^14^. Having first confirmed that Axin 2 expression and hence Wnt/β-cat signaling is upregulated following tooth damage we reasoned that addition of Wnt signaling agonists may provide an effective way to stimulate reparative dentine formation and thus restore lost dentine following caries removal with naturally-generated new dentine (Fig. S1). Numerous small molecule inhibitors of glycogen synthase kinase 3 (GSK3) have been developed and shown to efficiently upregulate Wnt activity in different experimental contexts and in one case, that of Tideglusib (NP-12, NP03112), are in clinical trials for the treatment of neurological disorders such as Alzheimers disease^15^,^16^,^17^,^18^,^19^,^20^,^21^. We tested the ability of three small molecule GSK3 inhibitors, BIO (2′Z,3′E)-6-Bromoindirubin-3′-oxime), CHIR99021(6-[[2-[[4-(2,4-Dichlorophenyl)-5-(5-methyl-1H-imidazol-2-yl)-2 pyrimidinyl]amino]ethyl]amino]-3-pyridinecarbonitrile) and Tideglusib (4-Benzyl-2-(naphthalen-1-yl)-[1,2,4]thiadiazolidine-3,5-dione) to stimulate tertiary dentine following experimentally induced pulp exposure^22^,^23^,^24^. As a delivery vehicle we used a commercially-available, clinically-approved collagen sponge, Kolspon.

## Results

### Effective concentrations and cytotoxicity testing

**Figure 1:**
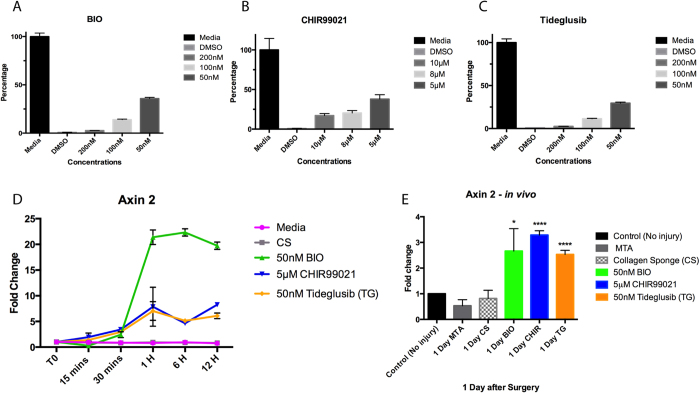
Drug titration and agonist activation of the Wnt pathway. MTT cytotoxity assay for (**A**) BIO, (**B**) CHIR99021, and (**C**) Tideglusib. (**D**) Axin2 qPCR for the *in vitro* assay with the 17IA4 cell line shows that when 50 nM BIO, 5 μm CHIR, and 50 nM Tideglusib are in the sponge, Wnt activity increases after 30 minutes of incubation and remains elevated. This elevation is not seen when just media or collagen sponge without the drug are incubated with the cells. (**E**) Axin2 qPCR for dental pulp cells collected either without injury or after one day of injury and capping with the conditions. BIO, CHIR and Tideglusib shows significant upregulation of Wnt activity when compared with control, MTA or collagen sponge. *P = 0.0365, ****P < 0.0001.

17IA4 mouse dental pulp cells were incubated with a range of concentrations of the three inhibitors and cytotoxicity analysed with the MTT assay after 24 h in culture (Fig. 1A–C)^25^,^26^. The highest concentration of inhibitor that was not cytotoxic was used in separate assays with the same cells and levels of Axin2 measured by qPCR in the first 24 h of culture. Increased Axin2 expression was observed after 30 mins and this reached a maximum after 1 hr (Fig. 1D). BIO induction of Axin2 expression was four fold greater than both CHIR99021 and Tideglusib, each of which showed similar levels of induction (Fig. 1D).

**Figure 2:**
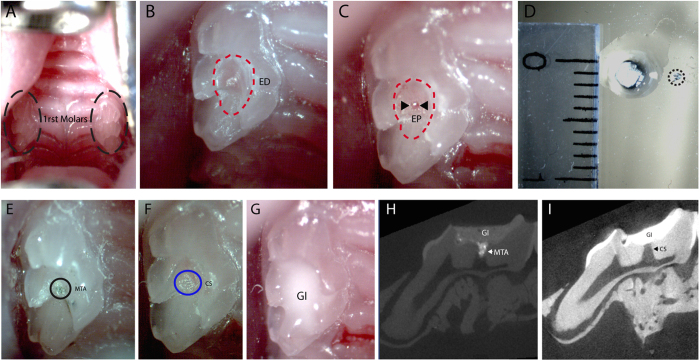
Injury and direct tooth capping. (**A**) photograph of upper first molars. (**B**) A 1/4 carbide burr cuts the tooth exposing the dentine until the roof of the pulp chamber (red dashed line). (**C**) Using a needle the dental pulp is exposed, indicated by the arrowheads. (**D**) The collagen sponge is soaked in drug and a small piece of it, indicated by the black dashed line, is removed for the direct capping. (**E**) The injury capped with MTA. (**F**) The sponge piece condensed inside the exposed pulp area. (**G**) The tooth is then sealed with glass ionomer until the date of collection. (**H**) MicroCT image right after capping showing the close contact of MTA (RO area indicated by arrow) with the dental pulp and the glass ionomer sealing. (**I**) MicroCT image right after capping showing the close contact of the collagen sponge (RL area indicated by arrow) with the dental pulp and the glass ionomer sealing. ED, exposed dentine; EP, exposed pulp; CS, collagen sponge; GI, glass ionomer; RO, radiopaque; RL, radiolucent.

To test the induction of Axin2 *in vivo*, experimental tooth damage was created, by drilling and making 0.13 mm holes in mouse maxillary first molars to expose the pulp (Fig. 2). Pieces of Kolspon were cut to size and soaked in solutions of the three inhibitors before being physically placed into the holes, in contact with the pulp. A glass ionomer cement was used to cover the sponge and protect the tooth (Fig. 2G). Treated teeth were removed after 24 h along with controls consisting of untreated teeth, MTA only and collagen sponge with no inhibitor. Pulp cells were extracted and tested for expression of Axin2 by qPCR (Fig. 1E). Expression of Axin2 was found to be 3 fold higher in inhibitor treated pulp cells when compared to controls (Fig. 1E). Significantly MTA showed no effect on Axin2 expression over controls suggesting current protocols do not lead to enhanced activation of Wnt signalling. After 5 days post treatment, Axin 2 expression levels were the same in with MTA and agonist treatments but these results were compounded by the fact that newly forming odotonblast-like cells express high levels of Axin 2 (Figs S1–2).

### Reparative dentine formation

**Figure 3:**
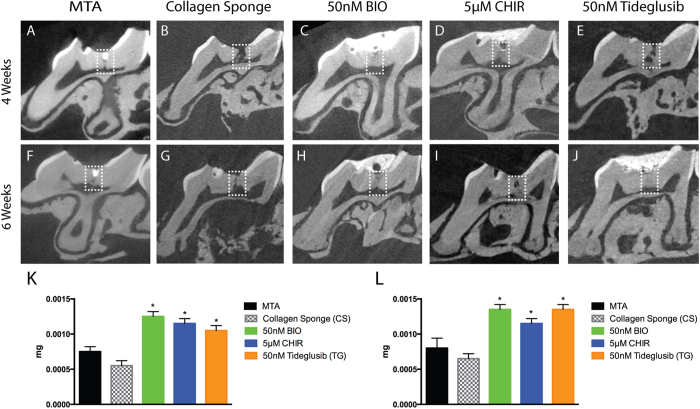
MicroCT analysis of mineral deposition. (**A**) MTA repair after 4 weeks, note the material (strong RO area at the injury site) at the injury site. (**B**) Collagen sponge repair after 4 weeks, spaced dentine formation at the injury site. (**C**) BIO, (**D**) CHIR, and (**E**) Tideglusib repairs show mature mineral at the injury site after 4 weeks. (**F**) MTA repair after 6 weeks still shows material at the injury site (strong RO area at the injury site). (**G**) Collagen sponge treatment shows injury mildly repaired. (**H**) BIO and (**I**) CHIR repair after 6 weeks displays injury site filled with mature dentine. (**J**) Tideglusib repair after 6 weeks shows mature reparative dentine formed at the injury site almost at the same radiopacity as the primary/secondary dentine. No external material is seen at the injury site after repair when teeth were treated with signalling modulators in collagen sponge. (**K**,**L**) 4 and 6 weeks, respectively, Mineral formation analysis at the injury site shows that teeth treated with small molecules form more mineral than when treated either with collagen sponge or MTA. 4 weeks BIO *P = 0.0101, 4 weeks CHIR *P = 0.0136, 4 weeks Tideglusib *P = 0.0194; 6 weeks BIO *P = 0.0101, 6 weeks CHIR *P = 0.0194, 6 weeks Tideglusib *P = 0.0101.

**Figure 4:**
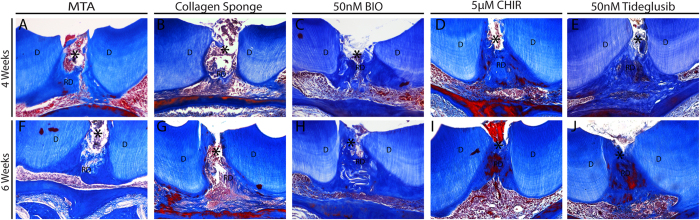
Histology of reparative dentine formation and pulp vitality. (**A**) 4 weeks MTA repair shows dentine formed underneath where the material was placed. (**B**) Collagen sponge shows sparse dentine formation in the dental pulp. (**C**) BIO, (**D**) CHIR, and (**E**) Tideglusib repairs show dense dentine formation at the injury site with vital pulp after 4 weeks. (**F**) 6 weeks MTA repair shows dentine formed underneath where the material was placed. (**G**) Collagen sponge repair shows little and immature dentine formed at the injury site after 6 weeks. (**H**) BIO treatment shows new mature dentine formed where the sponge was placed filling the injury site. (**I**) CHIR treatment shows mature new mature dentine formed where the sponge was placed filling the injury site. (**J**) Tideglusib treatment shows complete repair with vital dental pulp after 6 weeks.

Having established that the experimental model of tooth damage and pulp exposure provided a way of delivering small molecules that were able to affect pulp cell gene expression in a reproducible way, we used this method to examine the effect on the formation of reparative dentine. Maxillary molars were drilled and sponges inserted and left for 4–6 weeks before removal. Micro-computed tomographic (μCT) scanning was used to visualise and quantify mineral deposition at the drill site. Analysis at both 4 and 6 weeks revealed increased mineralisation with all three agonists when compared to controls with no obvious increases between 4 to 6 weeks (Fig. 3K,L). These increases were statistically significant for BIO, CHIR and Tideglusib both at 4 and 6 weeks. Overall the mineralisation with the inhibitors was on average 2 times greater than in the sponge alone control and 1.7 times greater than with MTA treatment. Following decalcification, sections were made through each molar and stained to reveal new dentine formation. The sections confirmed the μCT data showing that when teeth were treated with GSK-3 inhibitors, more reparative dentine was formed at the injury site than with collagen sponge or MTA (Fig. 4). Moreover, the new dentine formed with the new conditions presented as dense dentine localised centrally to the injury site, revealing no remaining collagen sponge where the dentine was formed. Interestingly, by 6 weeks of treatment, the reparative dentine secreted when teeth were treated with BIO, CHIR, and Tideglusib filled the whole injury site from occlusal to pulp chamber roof (Fig. 4H–J). Most importantly, dental pulp remained vital compared to controls consisting of exposed pulp with no capping, glass ionomer only showed no evidence of reparative dentine formation and severely hypoplastic pulp (Figs 4H–J; S3).

## Discussion

Modern dental practice for carious lesions aims to remove decay and restore tooth structure by using mineral aggregate filling materials. Preservation of undamaged dentine forms an integral part of this practice since maintenance of as much of the natural mineral as possible is deemed important for tooth vitality. Mineral aggregates such as MTA and Biodentine are reported to aid the formation of tertiary dentine, although the deposition of this dentine is not at the sites of damage but rather internal in the pulp space^27^,^28^,^29^. In addition the non-biodegradeable nature of these materials means that the full mineral volume is never restored. If a simple method can be developed that acts to enhance the natural processes of dentine restoration by stimulating tertiary dentine formation, then large injuries that would certainly lead the dental pulp to undergo necrosis could be repaired by enabling reparative dentine to be formed at the site of damage. The activation of Wnt/βCat signalling as a universal immediate-early response to tissue damage provides a potential route for enhancing natural repair by overstimulating this pathway^30^. Wnt/βcatenin signalling has thus emerged as a major target in tissue regeneration and repair and this pathway activity can be stimulated in a number of different ways. We chose small molecule agonists as a simple, cost-effective method that is supported by substantial existing experimental data and clinical use. In our damage model system we did not observe any effects of MTA on the enhancement of Wnt signalling activity and although it may be acting via other pathways it seems likely that any positive action on mineralisation is as result of providing mineral ions. We developed a method that uses an already clinically-approved biomaterial (collagen sponge – Kolspon) as a delivery vehicle for small molecule GSK-3 inhibitors that act as Wnt agonists. Both BIO and CHIR99021 have been extensively used experimentally to elevate Wnt activity while Tideglusib is in clinical trials for systemic use in the treatment of neurological disorders include Alzheimers disease^15^,^16^,^17^,^18^,^19^,^20^,^21^. Since upregulated Wnt activity in response to damage is an immediate early response we aimed to achieve rapid release of small molecule agonists and reasoned a sponge was the most effective way of ensuring this. All three agonists showed significantly increased mineralisation at the site of damage compared to the use of the sponge alone or MTA treatment. More significantly the localization of the reparative dentine formed indicated that with the treatments, the mineral replaced the biodegradable sponge and restored the cavity in the dentine made by the burr. With MTA the cavity remains permanently filled with mineral aggregate and this non-degradable material can only affect reparative dentine formation on the pulp chamber aspect.

An important consideration is the effect Wnt agonists may have following their release into the circulation. The small localised doses of these agonists used were effective at increasing the formation of reparative dentine to the extent that almost complete repair of the lesion was observed after 6 weeks. These doses are substantially lower that those used in clinical trials of Tideglusib where 500–1000 mg were delivered systemically daily for 26 weeks^20^. We used a maximum of 21 pg of Tideglusib on the sponges and thus even if 100% of the drug on the sponge is released within a few hours, the maximum systemic concentration, assuming all the drug enters the circulation, would be no more than 21 pg in 1.5 ml. Mouse blood volume is approximately 3000 times smaller than that of a human and thus the mouse dosage in the circulation is equivalent to 63 ng in the human circulation, or 1000 times lower than used in clinical trials. Extrapolating the size of a mouse first molar to that of a human suggests that an equivalent lesion would require around 10 times more reparative dentine formation and thus the anticipated concentrations of Tideglusib required for human tooth repair would be well below that already tested in clinical trials.

Small molecule Wnt signalling agonists delivered via a biodegradable collagen sponge provide an effective repair of experimentally-induced deep dental lesions by promotion of reparative dentine formation. The simplicity of this approach makes it ideally translatable into a clinical dental product for treatments requiring dentine restoration and pulp protection that are currently treated with non-organic cements.

## Materials and Methods

### Study design

The purpose of this study was to investigate whether natural dentine repair could be enhanced by stimulating the formation of reparative dentine. Adult CD1 wild-type mouse first molars were used and damage stimulated by controlling drilling (see below). The drugs of choice and vehicle were based on previous reports in the literature and also translational potential into a simple, cost effective dental therapy.

In order to evaluate the reparative capacity of the drugs, six to nine teeth were evaluated per time point and a sample size of 20–30 teeth per group was used for the *in vivo* gene expression assay. The reparative time points used in this study were in accordance with UK Home Office animal regulations. This study was not blind and sample selection was not random.

### Injury Protocol

All animals used in this study were handled in accordance to UK Home Office Regulations project license 70/7866 and personal license I6517C8EF. Experimental procedures were approved by the King’s College Ethical Review Process. The mice were anaesthetized with a solution made with Hypnorm (Fentanyl/fluanisone - VetaPharma Ltd.), sterile water and Hypnovel (Midazolam - Roche) in the ratio 1:2:1 at the rate of 10 ml/kg intraperitonially. A rounded carbide burr FG 1/4 coupled to a high speed hand piece was used to access the dentine of the mouse superior molar teeth. Once the burr cut exposed the dentine, a 30G needle was used to penetrate the pulp. In order to protect the pulp from external contamination and stimulate dentine repair, the injury was capped either with ProRoot Mineral Trioxide Aggregate (MTA) (Maillfer Dentsply), or Kolspon (Fish Collage Type 1- Eucare Ltd) alone, or in association with 50 nM BIO (SIGMA), 5 μM CHIR99021 (SIGMA), or 50 nM Tideglusib (SIGMA) dissolved and diluted in DMSO, in contact with the pulp. A layer of 3 M Ketac-Cem Radiopaque was used as a capping material to seal the injured site. The damage was performed on the two upper first molars. For incisor damage a fine needle was inserted through the mouse lower tooth into the pulp. Post-op the mice were given Vetergesic (Buprenorphine – Ceva) at the rate of 0.3 mg/kg by intraperitoneal injection as analgesic. The animals were sacrificed after 1 day, 4 weeks and 6 weeks.

### Cytotoxity assay

17IA4 cells were plated in 96 well plates at 20,000 cells/cm^2^ and incubated (37 °C, 5% CO_2_/95% air, 100% humidity) for 24 hrs using standard culture medium. Thereafter, the medium was replaced with conditioned (drugs + media) and control media (media alone) for another 24 hrs. (10 μl of drug in DMSO + 90 μl of media resulting on the following concentrations BIO: 200, 100, 50 nM; CHIR99021: 10, 8, 5 μM; Tideglusib: 200, 100, 50 nM). To determine the cell metabolic activity, MTT (3-(4,5-Dimethylthiazol-2-yl)-2,5- diphenyltetrazolium bromide, Sigma) was added to the controls and the conditioned media after 24 hrs. The resulting formazan product was then dissolved in 200 μl of dimethyl sulfoxide per well (DMSO, Sigma). A colorimetric plate reader (Thermo Multiskan Ascent 354 microplate reader) was used to read the absorbance at 540 nm with background subtraction at 630 nm.

### *In vitro* drug release

17IA4 cells were plated in 24-well plates and incubated (37 °C, 5% CO_2_/95% air, 100% humidity) for 24 h using standard culture medium. Falcon^TM^ cell culture inserts for use with 24-well plates (3 μm pore size) were placed in the wells carrying 96 mm^2^ Kolspon cubes either dry or soaked in 30 μl of the drug optimal concentration for 15 and 30 minutes, 1, 6, and 12 hours. The cells were collected with TRIzol and stored at −20 °C.

### Pulp collection

P21 mice had their superior first molars drilled according to the drilling protocol. Tooth pulp tissue was collected according to the experiment time course after injury. The superior first molars were extracted using a 21 G needle as an elevator to lift them from the alveolar bone and the extracted molars were kept in ice cold PBS. Using a 23 scalpel blade the molars were separated at the crown-root junction, so that the pulp chamber could be visualized. Using a 0.6 mm straight tip tweezer the pulp was gently scraped from the pulp chamber and the root canal. The pulp was then placed into cold Sigma RNAlater and stored at −80 °C.

### Q-PCR

RNA was extracted from the cells in culture with the drugs in after 15, and 30 mins, 1, 6, and 12 hours, and also from the dental pulp collected from CD1 P21 wild-type mice without injury (control) and 1 day after injury using TRIzol. (Thermo Fisher Scientific) as recommended by the manufacturer. The RNA was quantified using Nanodrop and reverse transcribed into cDNA. Beta-actin was used as housekeeping gene (Forward- GGCTGTATTCCCCTCCATCG, Reverse- CCAGTTGGTAACAATGCCTGT) and Axin2 was the read-out for Wnt pathway activity (Forward-TGACTCTCCTTCCAGATCCCA, Reverse-TGCCCACACTAGGCTGACA).

### MicroCT/mineral analysis

Mice upper molars were dissected and fixed with PFA 4% overnight and scanned using a Bruker Skyscan1272 micro-CT scanner. After scanning, Microview software programme (GE) was used for visualization and analysis. Two dimensional (2D) images were obtained from micro-CT cross-sectional images of superior first molar internal part, to evaluate if the drilling was successful and mineral formation. In order to assay tissue mineral content a ROI of X = 0.2 mm, Y = 0.4 mm, and Z = 0.2 mm was set as standard for all the samples and the mineral analysis was performed. The region measured comprised only of the injury site. ROI complete filled with mineral = 0.0017 mg.

### Histology

After 4 weeks decalcification in 19% EDTA the teeth were embedded in wax blocks and sectioned using 8 μm thickness. Sections were stained using Masson’s Trichrome.

### β-galactosidase staining

Teeth were fixed in 0.4% paraformaldehyde (PFA) for 24-hours at 4 °C, washed with PBS and decalcified in 19% EDTA pH 8 for 4-weeks. Teeth were immersed in 30% sucrose/PBS overnight at 4 °C before embedding in OCT (optimal cutting temperature) using dry ice and ethanol. Sections were cut at a thickness of 12-μm and were refixed (0.2% glutaraldehyde, 0.2% NP40, 5 mM EDTA, 2 mM MgCl_2_ in PBS) and washed in wash buffer (2 mM MgCl_2_, 0.02% sodium deoxycholate, 0.02% NP40 in PBS). β-galactosidase staining was visualized using a staining solution of 1 mg/ml X-gal substrate (Invitrogen) in wash buffer containing 5 mM potassium ferrocyanide andpotassium ferricyanide at 37 °C for 16-hour. Sections were dehydrated, cleared in Neo-Clear and mounted with Neo-Mount.

## Additional Information

**How to cite this article**: Neves, V. C. M. *et al*. Promotion of natural tooth repair by small molecule GSK3 antagonists. *Sci. Rep.* **7**, 39654; doi: 10.1038/srep39654 (2017).

**Publisher's note:** Springer Nature remains neutral with regard to jurisdictional claims in published maps and institutional affiliations.

## Supplementary Material

Supplementary Figures
