# Supplementary material for: Promotion of natural tooth repair by small molecule GSK3 antagonists
Source: Sci Rep. 2017 Jan 9;7:39654. doi: 10.1038/srep39654 (PMC5220443; doi:10.1038/srep39654)
Supplement: Supplementary Figures [file srep39654-s1.doc]

**Supplementary Information**

Promotion of natural tooth repair by small molecule GSK3 antagonists

Vitor Neves, Rebecca Babb, Dhivya Chandrasekaran and Paul T Sharpe

Department of Craniofacial Development and Stem Cell Biology

Dental Institute

Kings College London

Corresponding author: Paul T Sharpe, Department of Craniofacial Development and Stem Cell Biology, Floor 27, Dental Institute, Kings College London. paul.sharpe@kcl.ac.uk

**
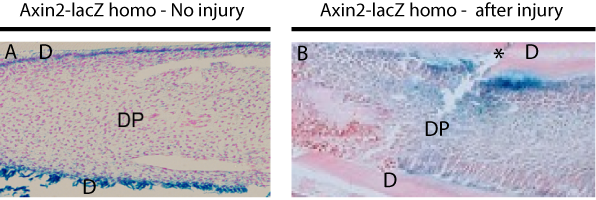
**

**Fig. S1. Axin2 levels increase at the injury site in mice teeth after damage**

Axin2 ^lacZ/lacZ^ reporter mice were used to visualise Wnt-βcatenin activity (12). (**A**) β-gal staining in the pulp of an incisor tooth without damage shows no Axin2 activity in the dental pulp. (**B**) Wnt activity is elevated 24h after damage. D, dentine; DP, dental pulp; *, damage site.


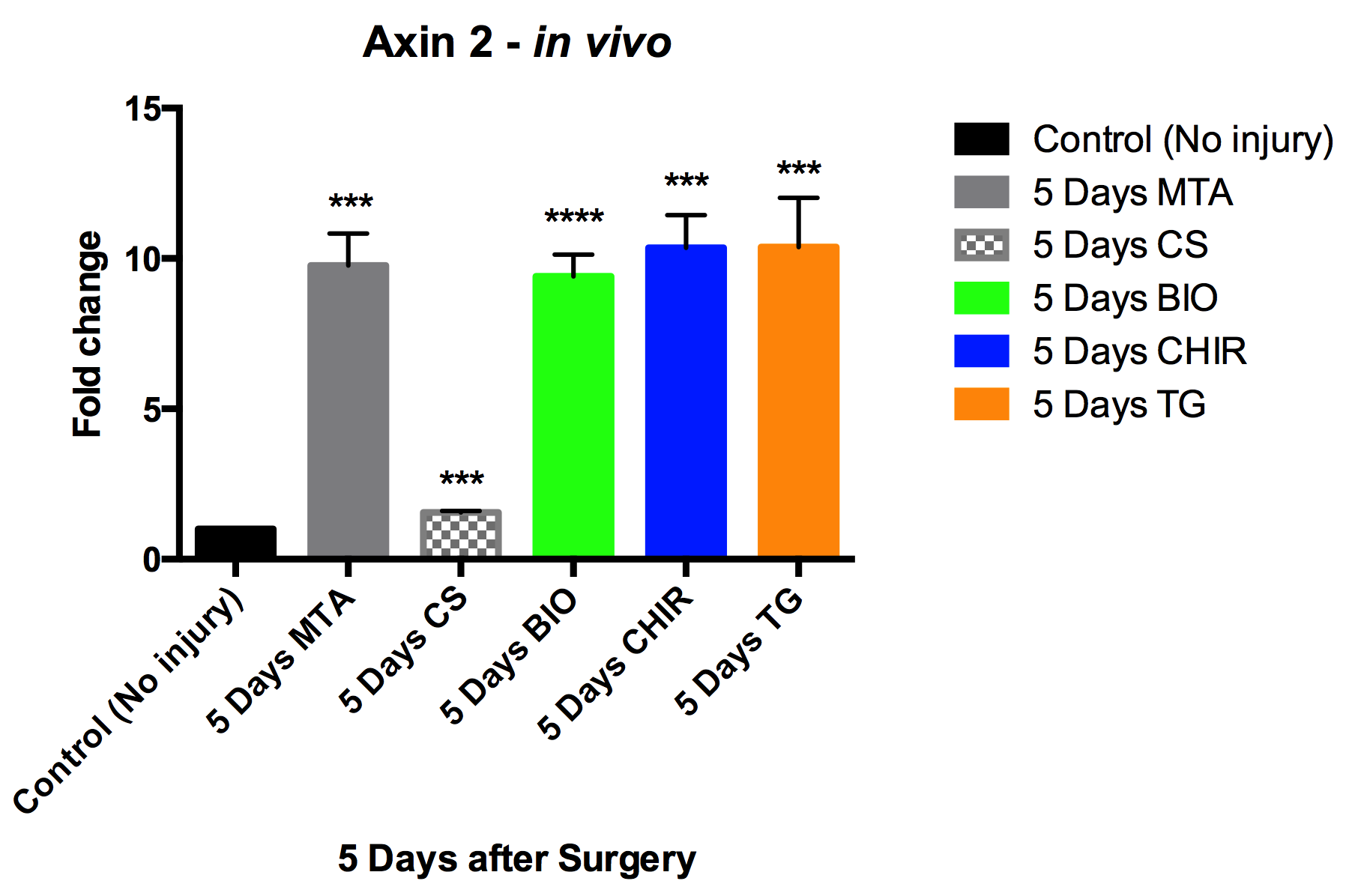


**Fig. S2. Axin2 expression levels 5 days after damage**

Axin2 qPCR for dental pulp tissue collected either without damage or 5 days after capping with the different treatments. MTA, BIO, CHIR and Tideglusib (TG) show similar levels of expression. MTA ****P*=0.0006; CS ****P*=0.0004; BIO *****P*<0.0001; CHIR ****P*=0.0001; TG ****P*=0.0006.


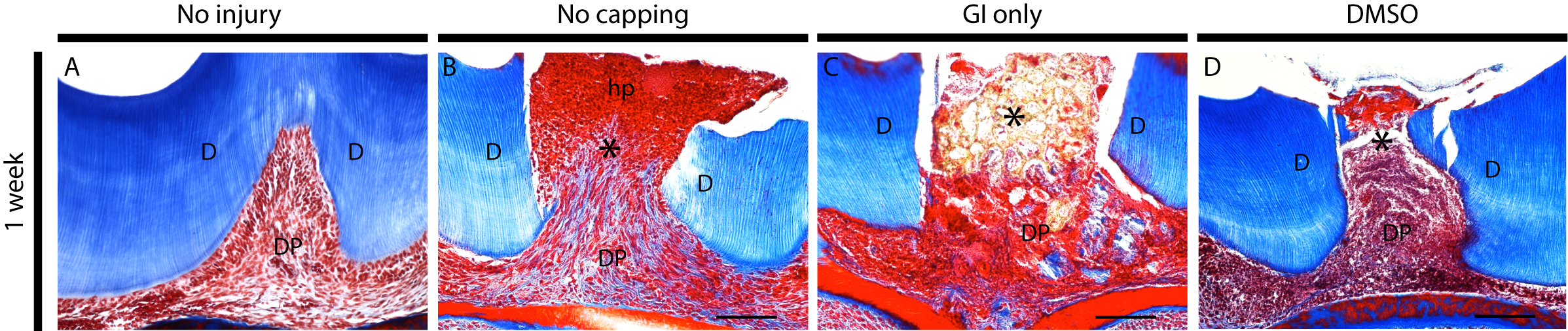


**Fig. S3. Comparison of different control conditions**

(**A**) Histology of molars without damage shows the normal dental pulp anatomy when vital. (**B**) Teeth left for one week without capping do not repair and the dental pulp becomes hyperplasic. (**C**) Molars capped with glass ionomer (GI) in direct contact with the dental pulp or the collagen sponge soaked only with DMSO (**D**), showed evidence of pulp necrosis D, dentine; DP, dental pulp; hp, hyperplasic tissue; *, damage site.
